# Comparison of two analytical platforms for quantification of neuroglial biomarkers in blood samples: a single molecule array and an electrochemiluminescence assay

**DOI:** 10.1038/s41598-026-56794-x

**Published:** 2026-06-30

**Authors:** Julia Aulin, Asma Al-Grety, Karl Sjölin, Ida Erngren, Anders Larsson, Anders Svenningsson, Kim Kultima, Joachim Burman

**Affiliations:** 1 Department of Medical Sciences, Cardiology, Uppsala University, Uppsala, Sweden; 2 Uppsala Clinical Research Center, Uppsala University, Uppsala, Sweden; 3 Department of Medical Sciences, Clinical Chemistry, Uppsala University, Uppsala, Sweden; 4 Department of Medical Sciences, Translational Neurology, Uppsala University, S-751 85 Uppsala, Sweden; 5 Department of Clinical Sciences, Karolinska Institutet Danderyd Hospital, Stockholm, Sweden

**Keywords:** Neurofilament light chain, Glial fibrillary acidic protein, Neuroglial biomarkers, Analytical platforms, Single-molecule array, Electrochemiluminescence assay, Biochemistry, Biological techniques, Biomarkers, Neurology, Neuroscience

## Abstract

Advances in ultrasensitive immunoassays have enabled reliable quantification of neuroglial biomarkers in blood, providing valuable insights into neurological disorders. However, cross-platform evaluations are necessary to ensure comparability and standardization. This study aimed to compare the analytical performance of a Single Molecule Array (Simoa) and an ultrasensitive electrochemiluminescence (ECL) assay for quantifying neurofilament light chain (NfL) and glial fibrillary acidic protein (GFAP) in human serum. Baseline serum samples from 174 participants in the RIFUND trial were analyzed in parallel on both platforms. Concentrations of NfL and GFAP were compared using Spearman correlation, Bland–Altman analyses, reproducibility assessments, dilution linearity, and cross-platform recovery. All samples were quantifiable on both platforms. NfL concentrations correlated strongly between methods (Spearman correlation *r* = 0.88, *p* < 0.0001), whereas GFAP correlated moderately (*r* = 0.77, *p* < 0.0001). Inter- and intra-assay coefficients of variation were comparable between platforms for both analytes. The relationship between the two assays could be described with the following equations: NfL_ECL_ = 7.63 × NfL_SIMOA_ + 1.71 and GFAP_ECL_ = 0.332 × GFAP_SIMOA_ + 4.22. Dilution linearity was excellent on both platforms (R^2^ > 0.99), although cross-platform recovery varied systematically across the analytical range. Both Simoa and ECL demonstrated strong analytical performance for neuroglial biomarker quantification in serum. Despite systematic differences in absolute concentrations, relative agreement was high, particularly for NfL. These findings highlight the need for platform harmonization and provide empirically derived conversion factors to support analytical comparability in research and clinical applications.

**Supplementary Information:**

The online version contains supplementary material available at 10.1038/s41598-026-56794-x.

## Introduction

Blood-based biochemical markers are widely used in clinical medicine to assess organ injury and function. In contrast, assessment of the central nervous system (CNS) has historically relied on imaging and cerebrospinal fluid (CSF) analyses in addition to the clinical examination. However, recent advancements in high-sensitive immunoassay technologies have enabled reliable detection of CNS-derived proteins in blood, offering new possibilities for less invasive and more accessible biochemical-based diagnostics.

The development of the Single Molecule Array (Simoa®) platform by Quanterix represented an important advancement in enabling detection of CNS-derived proteins in blood. Simoa is a digital immunoassay, where magnetic beads are coated with capture antibodies that bind to target analytes in biological fluids such as blood (plasma or serum) or CSF. These beads are then isolated in femtoliter-sized microwells, where individual binding events are detected as binary signals (“on” or “off”). This digital approach substantially increases analytical sensitivity and improves signal-to-noise, allowing detection of biomolecules at concentrations far below the detection limits of conventional ELISA methods^1^. Simoa also allows multiplex measurements, enabling simultaneous quantification of several biomarkers in the same sample. This capability has facilitated studies of low-abundance proteins in neurological disease. This feature has enabled its widespread use in biomarker research in biomarker discovery, neurodegenerative disease research, and precision medicine applications, where low-abundance proteins are of critical interest^2^.

In parallel, the Meso Scale Discovery (MSD®) electrochemiluminescence (ECL) platform has emerged as an alternative to ELISA-based immunoassays for detecting proteins, nucleic acids, and small molecules with high sensitivity and precision. The MSD® ECL assay is based on a sandwich immunoassay format where captured antibodies are immobilized on an electrode surface and detection antibodies conjugated to electrochemiluminescent labels (e.g., ruthenium) bind to the target analyte. Upon electrochemical stimulation, the ECL reaction generates a luminescent signal proportional to the analyte concentration, allowing sensitive and specific detection^3^. Compared with conventional immunoassays, ECL assays provide a broad dynamic range sub-picomolar detection capabilities, making them well-suited for multiplex applications. Recent advancements in MSD’s S-PLEX technology, incorporating TURBO-BOOST™ labelling technology, have enhanced sensitivity, reported to improve sensitivity by approximately 10- to 100-fold compared to standard ECL assays and facilitating detection at femtomolar concentrations^4,5^.

Despite these technological advancements, comparative studies evaluating the performance of Simoa and MSD ECL assays for neuroglial biomarker quantification in serum remain limited.

Neurofilament light (NfL) and glial fibrillary acidic protein (GFAP) are among the most studied blood-based biomarkers of neuroaxonal and astrocytic injury. In multiple sclerosis (MS), serum NfL has been examined in numerous studies and has been associated with inflammatory activity, neurodegeneration, and treatment response. GFAP has been proposed as a complementary marker reflecting astroglial pathology.

The introduction of highly sensitive immunoassay platforms has enabled reliable detection of these proteins in blood. The Simoa platform was one of the first technologies allowing quantification of NfL and GFAP in serum at low concentrations and has therefore been widely used in MS research. The MSD S-PLEX electrochemiluminescence platform represents an alternative high-sensitivity approach that has increasingly been adopted in biomarker studies.

Given the growing use of blood-based neuroglial biomarkers in both research and clinical studies, it is important to evaluate how different immunoassay platforms compare in terms of analytical performance and agreement.

This study aimed to compare two high-sensitivity immunoassay platforms, Quanterix Simoa and MSD S-PLEX ECL, for quantifying NfL and GFAP in human serum. By assessing correlation, reproducibility, and analytical performance, we sought to determine how these assays produce comparable results and to identify potential discrepancies that may impact their clinical and research applications. These findings may contribute to ongoing efforts in biomarker standardization and assay harmonization, ensuring the reliability of blood-based neuroglial biomarker measurements across different laboratories and study populations. Simoa and MSD S-PLEX assays are among the platforms most commonly used for measuring NfL and GFAP in blood. Comparing results obtained with these technologies is therefore relevant for studies that use these biomarkers in clinical research. To our knowledge, this is the first direct analytical comparison of serum GFAP and NfL concentrations measured using the Simoa and MSD-ECL platforms in MS patients and healthy donors. The study focuses on methodological comparability and analytical agreement rather than clinical outcome validation.

## Materials and methods

### Serum samples and participants

Serum samples from patients with multiple sclerosis were collected at baseline from participants enrolled in the Rituximab versus Fumarate in Newly Diagnosed Multiple Sclerosis (RIFUND-MS, ClinicalTrials.gov no. NCT02746744) trial. Demographic characteristics of the study cohort are summarized in Table 1. The details of the RIFUND-MS trial have been published previously^6^. Briefly, the RIFUND-MS was a phase III, rater-blinded, randomised trial performed at 17 Swedish hospitals. Adults aged 18–50 years with relapsing–remitting multiple sclerosis or clinically isolated syndrome, diagnosed within 10 years, and recent disease activity were included. Patients were randomised (1:1) to oral dimethyl fumarate or intravenous rituximab. Masked raters assessed relapses, disability, and MRI outcomes. The primary outcome was the proportion of participants with at least one relapse, analyzed using intention-to-treat methods^6^.

**Table 1:** Demographic characteristics of the study cohort. Distribution of participants by sex and age, presented as mean ± standard deviation (SD).

Category	Numbers	Mean age ± SD
Women	117 (67.2%)	37 ± 8.5
Men	57 (32.8%)	36 ± 7.1
Total	174	36 ± 8.1

Concentrations of NfL and GFAP were determined in baseline serum samples in paired baseline serum samples from 174 participants and analyzed on two analytical platforms, i.e., with the Simoa technique and with the ECL assay, before comparative performance evaluations were conducted. Only baseline samples collected prior to treatment initiation were included in the present analysis, and no separate control group was analyzed.

The study protocol was approved by the ethical review board in Stockholm (Dnr 2016/473-32) and the Swedish Medical Products Agency (EudraCT 2015-004,116-38). All participants provided written informed consent prior to inclusion, and the study was carried out in accordance with good clinical practice (GCP) and in accordance with the Declaration of Helsinki.

### Biochemical procedures

A detailed overview of the analytical workflow, including sample preparation and pre-dilution steps, is provided in Supplementary Table 1.

#### Simoa

For the Simoa assay, the commercial N2PB Kit (cat. no. 103520, lot no. 504131) was used with the Quanterix SR-X instrument, which is based on the Simoa® technology (Quanterix Corporation, Billerica. MA. USA). According to the manufacturer’s kit insert, SR-X assay data sheet, and lot-specific certificate of analysis (CoA), the analytical range for serum NfL was 1.60–1,800 ng/L and GFAP 4.15–36,000 ng/L. Analytical sensitivity, assay range, dilution recommendations, and control specifications were based on manufacturer-provided kit documentation and certificates of analysis. Detailed information regarding antibody epitopes and calibrator composition was not available in the public assay documentation and was therefore not included in the comparison.

In total, 174 samples were analyzed in duplicates across 12 plates. The assay plate was prepared using conical bottom plates provided by Quanterix. Eight standards; two quality controls (QC), one low (LQC) and one high (HQC); and samples were pipetted into the wells. Samples, controls, and standards were run in duplicates, with each replicate placed in separate wells. Samples and controls were diluted four times. Magnetic beads were vortexed for a minimum of 30 s and then poured into a clean reagent reservoir. A total of 20 μL of beads, followed by 20 μL of detector buffer containing protein stabilizers and biotinylated detector antibodies, were dispensed into each well. The plate was covered with a black lid and placed on an orbital shaker for incubation at 35 °C with shaking at 800 rpm for 30 min. After the initial incubation, the plate was placed on the washer, and the first wash was started. Following this, 100 µL of diluted streptavidin-ß-galactosidase (SBG) was dispensed into each well, and the second plate incubation at 35 °C with shaking at 800 rpm for 10 min started. Once the second incubation was done, the plate was placed on the washer, and the protocol was followed. After aspiration, the plate was removed from the magnet, the lid replaced, and the beads were resuspended offline on the orbital shaker at 800 rpm for one minute. The plate was then returned to the washer magnet, and the offline resuspension procedure was repeated again. Upon completion of the two Buffer B resuspension steps, final aspiration was performed and the plate was allowed to dry on the washer magnet for 10 min. Following the 10-min drying step, the plate containing pelleted beads was transferred onto the SR-X instrument, and the run was initiated.

#### ECL

The commercial S-PLEX Neurology Panel 1 Kit (cat. no. K15639S), which is based on ultrasensitive ECL technology, was used as a comparison method. The protocol, designed for the MESO SECTOR S 600 instrument (Meso Scale Diagnostics, Rockville, MD, USA), involved a multi-step incubation and detection process described below. According to the manufacturer’s package insert and lot-specific certificate of analysis (CoA), the analytical range for serum NfL was 8.48–1,400 ng/L and GFAP 1.34–450 ng/L. Detailed information on antibody epitopes was not available in the public documentation and was therefore not included in the comparison. Analytical performance characteristics and dilution protocols were based on manufacturer-provided assay documentation.

A total of 174 samples were analyzed in duplicates across 6 plates. The assay plate was prewashed three times with 150 μL of 1 × MSD Wash Buffer using an automated plate washer. A biotinylated capture antibody solution was prepared by mixing 4 × Coating Solution with Diluent 100 in a 1:4 ratio. Of this solution, 50 µL was pipetted into each well. The plate was then sealed and incubated at room temperature on an orbital shaker set at 700 rpm for one hour. After washing the plate, 25 μL of blocking solution was dispensed into each well, followed by 25 μL calibrators and a diluted sample. Calibrators were prepared as a seven-point curve using four-fold serial dilutions starting from the stock calibrator, with a zero-calibrator as the blank. Serum samples were diluted twice. The plate was sealed and incubated on the orbital shaker at 700 rpm for 1.5 h at a temperature of 27 °C. After the incubation, the plate was washed three times with 1 × MSD Wash Buffer. A TURBO-BOOST antibody solution was prepared, and 50 µL of this solution was added to each well; the plate was then sealed and incubated for one hour on the orbital shaker at 700 rpm at 27 °C. After the 1-h incubation, an enhance solution was prepared and 50 µL of the enhance solution was added to each well after washing the plate. The plate was incubated for 30 min on the orbital shaker at 700 rpm at room temperature. After washing the incubated plate, 50 µL of TURBO-TAG detection solution was added to each well. The plate was then sealed and incubated for 1 h at 27 °C with shaking at 700 rpm. Following the final incubation, the plate was washed three times with 1 × MSD Wash Buffer using the low-flow dispense setting on the automated washer. MSD GOLD Read Buffer B was added to each well at a volume of 150 μL, and the plate was immediately transferred to the MESO SECTOR S 600 instrument for analysis. An instrument-specific protocol was preloaded into the MESO QuickPlex software. Calibration data were adjusted based on the lot-specific Certificate of Analysis (CoA) included with the kit.

Recovery was evaluated by measuring the calibrators provided with each assay on the opposite platform. The measured concentrations were compared with the nominal values stated in the certificate of analysis, and recovery was calculated as measured/nominal × 100%.

### Statistical analysis

All analyses were performed using GraphPad PRISM v 10.4.1 (GraphPad Software, La Jolla, CA). No formal sample size calculation was performed, the study included 174 participants based on availability of samples from the RIFUND-MS trial. No formal test of outliers was conducted and all data points were included in the analyses. Medians with interquartile range (IQR) were used to summarize data. Correlations were described with Spearman’s r and classified according to the British Medical Journal guidelines^7^. Passing-Bablok regression was performed to assess agreement between methods while accounting for measurement error in both variables.

Analytical performance was evaluated by assessing inter- and intra-assay precision, correlation between platforms, dilution linearity, and cross-platform recovery within the manufacturer-specified analytical ranges. Inter-assay precision was assessed using different materials on the two platforms: manufacturer-provided control material for Simoa and pooled human serum for MSD ECL. As these materials are not equivalent, the resulting precision estimates reflect assay performance within each platform rather than a directly comparable head-to-head evaluation using matched biological samples. Accordingly, these analyses should be interpreted as descriptive of assay repeatability under the study conditions. The study was designed as a comparative evaluation of two commercially available assays rather than a formal analytical validation according to CLSI guidelines.

The manufacturer-specified analytical measurement ranges, including the lower and upper limits of quantification, were used to ensure that all reported concentrations fell within the validated range of each platform.

## Results

The serum samples had a median NfL concentration of 9.7 (IQR 7.6–16) ng/L on the Simoa assay and 75 (IQR 61–120) ng/L on the ECL assay. The median GFAP concentrations were 66 (IQR 48–88) ng/L on the Simoa assay and 25 (IQR 19–33) ng/L on the ECL assay. All samples fell within the analytical range provided by each manufacturer.

### Coefficients of variation

To assess the interassay coefficient of variation (CV) for the Simoa assay, low and high control materials provided by the manufacturer (lyophilized antigen with protein stabilizers) were run on each plate. The interassay CV for NfL was 6.8% for the low controls (mean 7.1 ± 0.48 ng/mL) and 8.1% for the high controls (mean 500 ± 40 ng/L). The interassay CV for GFAP was 9.2% for the low controls (mean 100 ± 9.5 ng/L) and 12% for the high controls (mean 11,000 ± 1,300 ng/L).

To assess the interassay CV for the ECL assay, we used a pool of serum samples run on each plate. The interassay CV for NfL was 8.8% (mean 80 ± 7.0 ng/L). The interassay CV for GFAP was 4.7% (mean 24 ± 1.1 ng/L).

The pooled intraassay CV was 5.9% for NfL and 8.8% for GFAP with the Simoa. The pooled intraassay CV was 4.2% for NfL and 1.7% for GFAP with the ECL assay. Intra-assay CVs were calculated using pooled human serum samples.

### Correlation between the assays

**Fig. 1:**
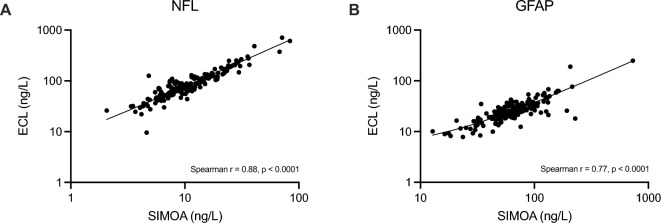
Correlations between Simoa and ECL assay measurements. Correlation between serum concentrations of neurofilament light chain (NfL) (**A**) and glial fibrillary acidic protein (GFAP) (**B**) measured on the Quanterix Simoa platform and the MSD S-PLEX ECL platform (*n* = 174). Each point represents one patient sample. Spearman correlation coefficients are indicated in the panels. NfL measurements showed very strong cross-platform correlation, while GFAP displayed a strong but significant correlation.

**Fig. 2:**
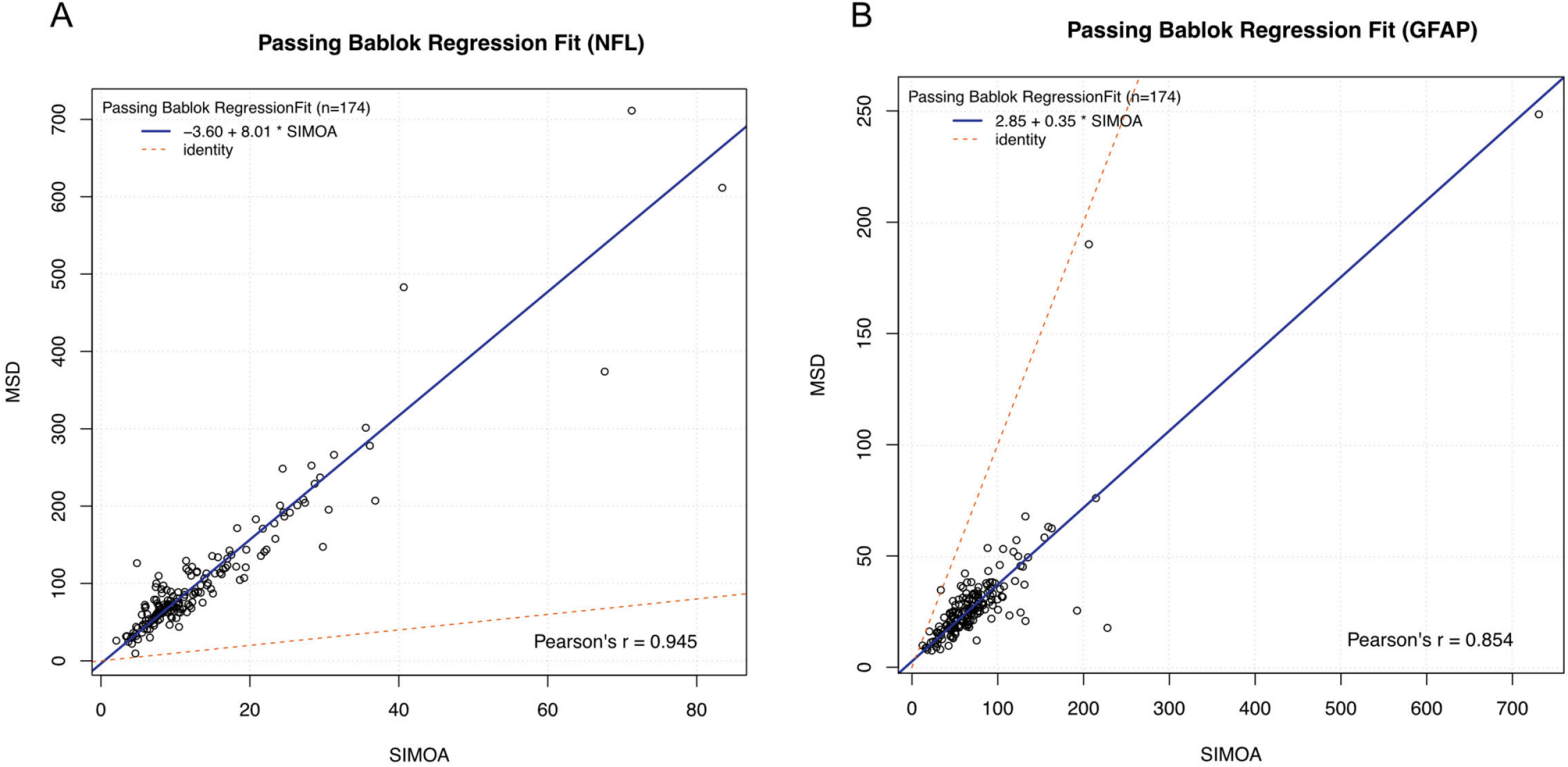
Passing–Bablok regression analysis of NfL and GFAP concentrations measured using the Simoa and MSD platforms. Passing–Bablok regression was used to assess agreement between platforms. A strong correlation was observed for NfL (*r* = 0.945), whereas GFAP showed a moderate correlation (*r* = 0.854). Deviations from the identity line indicate systematic differences between methods.

Serum NfL concentrations showed a very strong correlation between the two analytical platforms (Spearman *r* = 0.88, *p* < 0.0001, Fig. 1A), and moderate for serum GFAP (Spearman *r* = 0.77, *p* < 0.0001, Fig. 1B). Passing–Bablok regression was performed to account for measurement error in both methods (Fig. 2). The analysis demonstrated a strong linear relationship for NfL and a moderate relationship for GFAP, with a systematic deviation from the identity line, indicating differences in absolute concentrations between platforms. A linear regression analysis was made for descriptive purposes, and the relationships between the two assays were described by the equations NfL_ECL_ = 7.63 × NfL_SIMOA_ + 1.71 and GFAP_ECL_ = 0.332 × GFAP_SIMOA_ + 4.22. Rearranging these gives the reverse equations that estimate SIMOA values from ECL measurements: NfL_SIMOA_ = 0.131 × NfL_ECL _− 0.224 and GFAP_SIMOA_ = 3.01 × GFAP_ECL _− 12.7. Based on this analysis, we established a conversion factor of 7.63 for NfL and 0.332 for GFAP.

### Comparison between the assays

**Fig. 3:**
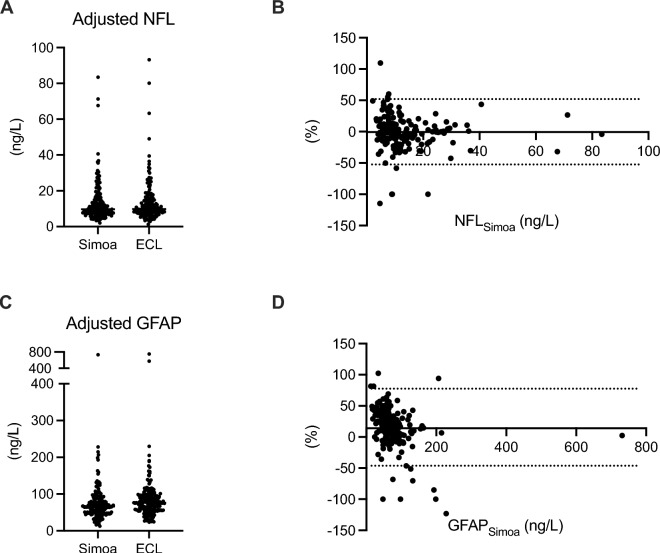
Cross-platform comparison of measured and converted concentrations. Comparison of converted neurofilament light chain (NfL) and glial fibrillary acidic protein (GFAP) concentrations measured using Simoa and ECL assays (*n* = 174). Linear regression analysis was used to derive conversion factors of 7.63 for NFL and 0.332 for GFAP, allowing ECL values to be converted to align with Simoa measurements. Panels (**A**)and (**C**) display paired serum concentrations of NfL and GFAP, respectively, measured by both platforms. Each dot represents an individual sample. Panels (**B**) and (**D**) show Bland–Altman plots of the percentage differences between conveted ECL and Simoa values plotted against the Simoa concentrations (reference method). The solid horizontal line indicates the mean bias, and the dashed lines represent the 95% limits of agreement (LoA).

The serum concentrations of NfL and GFAP obtained with the ECL assay were converted to equivalent values on the Simoa assay using the conversion factor. The distribution of the NfL and GFAP concentrations obtained with the Simoa assay and the adjusted values of the concentrations obtained with the ECL assay after conversion, are shown in Fig. 3A and C. Agreement between the two methods was further assessed using Bland–Altman analysis (Fig. 3B and D). The analysis demonstrated a systematic bias between platforms, with MSD generally yielding higher concentrations for NfL and lower concentrations for GFAP compared with Simoa. The wide limits of agreement indicate considerable variability between methods, particularly at higher concentrations, suggesting concentration-dependent differences between platforms.

### Dilution linearity and sensitivity

**Fig. 4:**
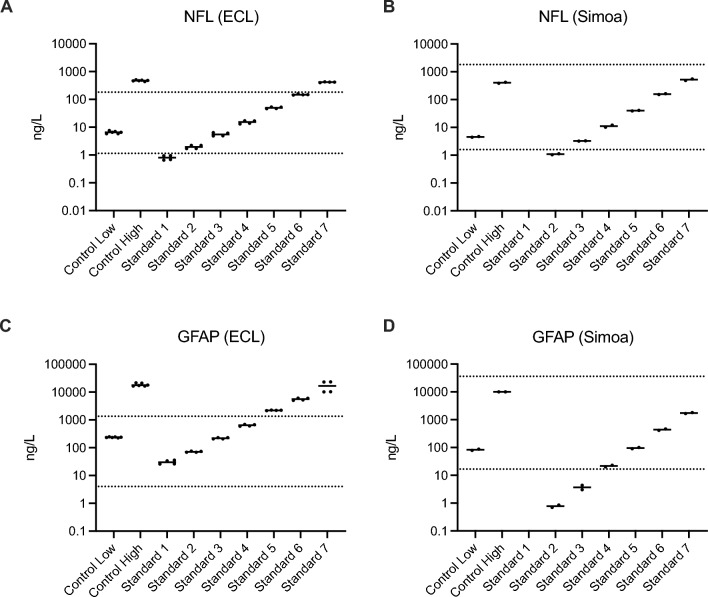
Reproducibility of calibrator and control measurements across platforms. Concentrations of controls and standards cross-platform. The dashed lines indicate the analytical range provided by the manufacturer. The measured concentrations of the control samples (low & high) provided by Quanterix when run on the MSD platform (**A** & **C**) and the Simoa platform (**B** & **D**) are shown to the left in each diagram. Concentrations of the standards used for establishing the standard curve on the other instrument are shown on the right.

To assess linearity, the standards from the ECL were run with the Simoa assay and vice versa. Five out of seven Simoa NfL standards and four out of seven Simoa GFAP standards fell within the analytical range of the ECL assay (Fig. 4A and C).

Five out of seven ECL NfL standards and four out of seven ECL GFAP standards fell within the analytical range of the Simoa assay (Fig. 4B and D). However, the lowest ECL standards could not be detected with the Simoa assay but were also outside the analytical range. All the standards displayed a high dilution linearity within the analytical range with R^2^ > 0.99 for all analytes.

**Fig. 5:**
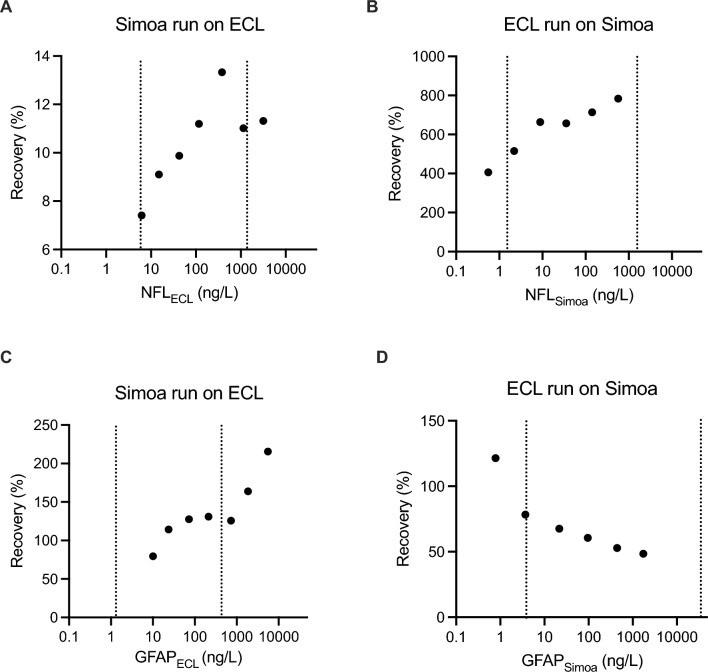
Cross-platform recovery of NFL and GFAP calibrators. (**A**, **C**) Simoa calibrators measured on the ECL platform and (**B**, **D**) ECL calibrators measured on the Simoa platform. The x-axis shows the measured concentration on the receiving platform (ng/L; log scale). The y-axis shows recovery, calculated as (measured concentration ÷ nominal concentration on the calibrator’s native platform) × 100%. Points show means of replicate measurements. For NfL, recoveries are consistently below 100% when Simoa calibrators are measured on the ECL platform and above 100% in the reverse direction, with a magnitude that approximates the cross-platform scaling factor derived from patient samples. This pattern indicates that the absolute differences are largely driven by calibrator value assignment, rather than random variability. For GFAP, recovery varies with concentration, indicating proportional bias rather than a constant shift. Together, these data clarify that absolute value discrepancies arise from a combination of calibrator value assignment and assay-intrinsic differences.

To further investigate the source of differences between the two platforms, we compared the measured concentrations of each standard with its nominal value and calculated the recovery. As expected, recovery of native measurements on their respective platforms was excellent, with mean recovery of 99.1–101% with Simoa and 100–102% with ECL. Cross-platform measurements showed considerable differences in recovery both between platforms, but also depending on where in the analytical range the measurements were made (Fig. 5A–D).

## Discussion

In this study we compared two ultrasensitive immunoassay platforms for quantification of NfL and GFAP in human serum. We demonstrate strong cross-platform concordance for NfL and moderate concordance for GFAP, while also documenting systematic differences in absolute concentrations. These findings align with recent cross-platform studies and highlight the need for improved calibration and standardization between assays. A more robust long-term solution will likely require the development of certified and commutable reference materials and higher-order reference procedures. At present, such materials are not yet routinely available for serum NfL and GFAP, and inter-assay comparability therefore remains dependent on manufacturer-specific calibration.

While the values obtained with the Simoa (Quanterix) and ECL (MSD) assays correlated strongly, the absolute concentrations differed substantially. This is expected because two immunoassays designed to measure the same antigen may yield different results due to structural differences in the antigen that affect how antibodies recognize and bind to it^8,9^. Both NfL and GFAP are present in very low concentrations in vivo, making it challenging to prepare sufficient amounts of native proteins for use as immunogens, calibrators, and controls^9,10^. Consequently, synthetic peptides or recombinant proteins are typically used, which may differ in glycosylation compared to native human proteins^8–10^.

In addition, circulating NfL and GFAP concentrations are known to be influenced by biological factors such as age and neurological comorbidities. Pre-analytical factors such as sample handling, storage conditions, and freeze–thaw cycles may also influence measured concentrations. Both NfL and GFAP are glycosylated, and the location, extent, and pattern of glycosylation can vary between individuals, disease states, or even sample types^8,10^. These sugar moieties can block antibody binding sites (epitopes) or alter protein conformation, thereby influencing antigen detectability depending on the antibody specificity of each assay^8,10^. In addition to glycosylation, differences in conformational stability, degradation state, and epitope availability can all affect how antibodies recognize the target protein. As a result, two assays that are both described as targeting “the same” protein may in practice detect different forms or fractions, leading to systematic differences in measured concentrations.

An important limitation of the present comparison is the lack of full transparency regarding antibody epitopes and calibrator structures for the commercial assays, as manufacturers consider detailed epitope information proprietary. Consequently, it cannot be concluded that the two platforms quantify identical circulating proteoforms of NfL and GFAP, even when the overall correlation is high. Furthermore, in the absence of universally accepted reference standards composed of native, fully characterized human proteins, it is not possible to determine which assay provides the more analytically accurate absolute concentration. Results should therefore be interpreted within the context of each assay’s calibration system and analytical characteristics.

Certified and commutable reference materials for serum NfL and GFAP are currently not routinely available. Ongoing harmonization initiatives may improve comparability between platforms and facilitate future standardization of blood-based biomarker measurements.

Our findings align with the recent cross-platform comparison by Sheth et al., who assessed five immunoassays for NfL across blood and CSF and reported very strong correlations but systematic differences in absolute values. Their work underscores that NfL values are not directly interchangeable across methods without harmonization^11^. Similarly, we observed high concordance between Simoa and ECL for relative NfL trends, with weaker agreement for GFAP. Overall performance in terms of reproducibility and dilution linearity was high for both platforms; inter-assay and intra-assay CVs were similar and dilution linearity was excellent (R^2^ > 0.99). Importantly, the recovery analysis revealed that the relationship between measurements differed across the analytical range. The conversion factors derived in this study should therefore be regarded as approximate and applicable only within the restricted concentration range represented in this dataset. These findings indicate that harmonization between platforms is concentration-dependent, and the conversion equations should therefore not be extrapolated beyond the studied interval. Simoa has long been regarded as the benchmark for ultrasensitive biomarker detection, particularly in Alzheimer’s disease research^12^. Previous studies have demonstrated that Simoa offers up to a 20-fold increase in sensitivity compared to earlier generations of MSD’s ECL assays^13^. However, recent improvements in ECL technology, most notably with the S-PLEX platform, have reduced this sensitivity gap, making MSD’s ultrasensitive assays a viable alternative. Studies comparing Simoa and MSD S-PLEX for cytokine and neuroinflammatory biomarker measurements have reported similar levels of sensitivity and specificity^4,14^, reinforcing the credibility of ECL assays for applications requiring high analytical sensitivity. In addition, a study investigating NfL quantification in Alzheimer’s disease reported a high correlation between Simoa and ECL measurements across 72 participants^15^, further supporting the feasibility of using either platform for neuroglial biomarker quantification.

This study has several limitations. First, the study was designed as a comparative evaluation of two commercially available assays rather than a formal analytical validation according to CLSI or other regulatory guidelines. Second, analyses were performed using the Simoa SR-X instrument, which is generally considered less analytically sensitive than the HD-X platform, commonly used in larger biomarker studies. Third, the cohort consisted exclusively of individuals with MS, and applicability to other populations requires further validation. Fourth, the conversion factors derived here are applicable only within the concentration ranges observed in this study. Fifth, dilution experiments using biological samples were not performed, as available serum material did not allow for such analyses across the required concentration range. In addition, precision and dilution-related assessments were not fully performed using matched biological matrices across both platforms. Manufacturer-provided control materials and assay calibrators prepared in buffer do not fully mimic native serum and should therefore be interpreted as descriptive of assay performance within each platform rather than as a direct head-to-head comparison under identical biological conditions.

## Conclusions

Our results show that both Simoa and ECL platforms can be used to quantify neuroglial biomarkers in human serum with good analytical performance. In particular, the strong correlation and reproducibility observed for NfL support the use of either platform in biomarker research. Advances in the MSD S-PLEX technology have substantially narrowed the historical sensitivity advantage of Simoa, positioning ECL assays as credible alternatives to digital immunoassays. As blood-based biomarkers become increasingly important for diagnosing and monitoring neurological disorders, cross-platform standardization will be essential to ensure consistent and clinically interpretable results. The conversion factors generated here provide an initial framework for such harmonization and represent an important step toward broader comparability of neuroglial biomarker measurements across laboratories and platforms.

## Supplementary Information

Below is the link to the electronic supplementary material.

Supplementary Material.

## Data Availability

The data supporting this article will be shared at a reasonable request by the corresponding author.

## Abbreviations

CNS: Central nervous system
CoA: Certificate of analysis
CSF: Cerebrospinal fluid
CV: Coefficient of variation
ECL: Electrochemiluminescence
GFAP: Glial fibrillary acidic protein
HQC: High-quality control
LLOD: Lower limit of detection
LQC: Low-quality control
MS: Multiple sclerosis
NfL: Neurofilament light chain
Simoa: Single molecule array
RRID: Research resource identifier (scicrunch.org)
QC: Quality control
