# Supplementary material for: Comparison of two analytical platforms for quantification of neuroglial biomarkers in blood samples: a single molecule array and an electrochemiluminescence assay
Source: Sci Rep. 2026 Jun 30;16:19939. doi: 10.1038/s41598-026-56794-x (PMC13319466; doi:10.1038/s41598-026-56794-x)
Supplement: Supplementary file 1 — Supplementary Material. [file 41598_2026_56794_MOESM1_ESM.pdf]

Supplementary

Table 1. Overview of analytical workflow and assay conditions for Simoa and MSD platforms

Detailed overview of the analytical workflow for the Simoa Neuro 2-Plex B (SR-X) and MSD S-PLEX Neurology Panel 1 assays. The table summarizes assay principles, sample handling procedures, pre-dilution steps, sample volumes, and key assay conditions for serum measurements of GFAP and NfL.

Parameter	***Simoa Neuro 2-Plex B (SR-X)***	***MSD S-PLEX Neurology Panel 1***
Assay principle	*2-step digital single molecule array immunoassay*	*Sandwich electrochemiluminescence immunoassay (S-PLEX)*
Analytes measured	*GFAP, NF-light*	*GFAP, Neurofilament L*
Validated sample type	*Human serum*	*Human serum*
Pre-analytical handling	*Thaw to RT, mix thoroughly, centrifuge prior to assay*	*Thaw on ice/2–8°C, centrifuge after thawing*
Sample volume before dilution	*25 µL serum (in-plate dilution)*	*30 µL serum (2-fold dilution example)*
Dilution factor (serum)	*4× dilution*	*2-fold dilution*
Total diluted sample volume	*100 µL (25 µL serum + 75 µL diluent)*	*60 µL (30 µL serum + 30 µL Diluent 64)*
Diluted sample volume added per well	*100 µL per replicate well*	*25 µL diluted sample per well*
Calibration curve fit	*4-parameter logistic (4PL), 1/y² weighting*	*4-parameter logistic (4PL), 1/y² weighting*
Key incubation conditions	*30 min at 35°C + 10 min at 35°C*	*1.5 h (22–27°C) + 1 h detection at 27°C*
Detection system	*Streptavidin-β-galactosidase, digital bead counting*	*TURBO-BOOST/TURBO-TAG electrochemiluminescence*
Instrument	*Quanterix SR-X*	*MSD SECTOR / QuickPlex*
